# Lactoferrin-Conjugated Nanoparticles as New Antivirals

**DOI:** 10.3390/pharmaceutics14091862

**Published:** 2022-09-03

**Authors:** Malgorzata Krzyzowska, Martyna Janicka, Emilia Tomaszewska, Katarzyna Ranoszek-Soliwoda, Grzegorz Celichowski, Jarosław Grobelny, Pawel Szymanski

**Affiliations:** 1 Military Institute of Hygiene and Epidemiology, Kozielska 4, 01-163 Warsaw, Poland; 2 Division of Microbiology, Department of Preclinical Sciences, Institute of Veterinary Medicine, Warsaw University of Life Sciences, 02-786 Warsaw, Poland; 3 Department of Materials Technology and Chemistry, Faculty of Chemistry, University of Lodz, Pomorska 163 St., 90-236 Lodz, Poland; 4 Department of Pharmaceutical Chemistry, Drug Analyses and Radiopharmacy, Faculty of Pharmacy, Medical University of Lodz, Muszynskiego 1, 90-151 Lodz, Poland

**Keywords:** viruses, lactoferrin, nanoparticles

## Abstract

Lactoferrin is an iron-binding glycoprotein with multiple functions in the body. Its activity against a broad spectrum of both DNA and RNA viruses as well as the ability to modulate immune responses have made it of interest in the pharmaceutical and food industries. The mechanisms of its antiviral activity include direct binding to the viruses or its receptors or the upregulation of antiviral responses by the immune system. Recently, much effort has been devoted to the use of nanotechnology in the development of new antivirals. In this review, we focus on describing the antiviral mechanisms of lactoferrin and the possible use of nanotechnology to construct safe and effective new antiviral drugs.

## 1. Introduction

Lactoferrin (LF) is a multifunctional glycoprotein present in external secretions, such as colostrum, milk, tears, nasal and bronchial secretions, saliva, bile and pancreatic secretions, urine, seminal and vaginal fluids, and has multiple functions, including as a component of the innate immune response [1,2]. Human lactoferrin (hLF) is constitutively synthetized and secreted by glandular epithelial cells at different epithelial surfaces [1,2]. Moreover, inflammatory processes that recruit neutrophils regulate hLF concentration, since neutrophils secrete secondary granules containing hLF. Lactoferrin belongs to the family of transferrins, and includes approximately 700 amino acids (80 kD) folded into two globular lobes with an α-helix as a linker. LF binds two ferric ions with high affinity, but it can also bind Cu2+, Zn2+ and Mn2+ ions [1,2]. LF has been shown to perform antioxidant, anticancer and anti-inflammatory activities, together with a broad antibacterial, antifungal and virucidal activity [1,2]. 

LF exists in an iron-free apo-lactoferrin (apoLF) form, while upon iron binding, LF undergoes large conformational changes in which hololactoferrin (hoLF) transforms into either an open or closed state [1,2,3]. Depending on the origin, LF can undergo glycosylation at three sites in humans (Asn-137, Asn-478 and Asn-623) or at five (Asn-233, Asn-368, Asn-476, Asn476, Asn545) in bovine lactoferrin (bLF), being mostly exposed on the outer surface of the molecule [3,4]. The nature and the location of the glycosylation does not influence lactoferrin’s ability to bind iron, but the specific and varied patterns of glycosylation influences its antimicrobial and immunomodulatory activities as lactoferrin glycan modifications have been described to be participate in different cellular pathways, including cell adhesion and receptor activation [3,4]. Human lactoferrin (hLF) shows a high sequence similarity with bovine lactoferrin (bLF), and displays similar biocidal activity against bacteria, viruses and fungi, as well as immunomodulatory and anti-inflammatory activities [2,5]. The commercial preparations of bLF were recognized as a safe substance (GRAS) by the Food and Drug Administration (FDA, Silver Spring, MD, USA) and are distributed by many companies in large quantities; therefore, the majority of the in vitro and in vivo studies have been using commercial bLF. Recently, recombinant human lactoferrin has also become available. 

## 2. Lactoferrin as Immunomodulator

Some authors suggest that, in a broader sense, LF may be classified as an “alarmin”, a small peptide released from neutrophils upon infection [6] and providing support for further immune reactions [7]. Lactoferrin shows the ability to bind to pathogen-associated molecular patterns (PAMPs) present various pathogens, mainly recognized by Toll-like receptors (TLRs) [7]. Depending on the pattern of glycosylation, lactoferrin can bind to different receptors present on both of epithelial and immune cells, including CD14 [8], LDL receptor-related protein-1 (LRP-1/CD91) [9], Toll-like receptor 2 and 4 (TLR4) [10] cytokine receptor 4 (CXCR4) [11], intelectin-1 (omentin-1) [12], dendritic cell-specific intercellular adhesion molecule-3-grabbing non-integrin on antigen-presenting cells (APCs) [13], Siglec-1 (CD169/sialoadhesin) [14] and nucleolin [15]. Most of these receptors bind other ligands, but lactoferrin also possesses its “own” receptor called lactoferrin receptor/LRP-1/CD91/apoE receptor or the chyclomicron remnant receptor [16]. ApoE is found in many tissues and different cell types, including intestinal epithelial cell lymphocytes, fibroblasts, neurons, hepatocytes and endothelial cells [16]. Some of these receptors may potentially endocyte lactoferrin and trigger cell signaling or target lactoferrin to the nucleus where it could act as a transcriptional activator or transactivator, which further adds to the multiplicity of roles played by LF. 

Upon infection, neutrophils release chromatin fibers, known as neutrophil extracellular traps (NETs), together with high amounts of elastase, myeloperoxidase (MPO) and lactoferrin—one million of neutrophils can release up to 15 μg of LF [17]. The role of NETs, together with excreted enzymes and peptides, is to trap and kill mostly bacteria [15]. However, some authors showed that LF can actually suppress the release of NETs during inflammation, thus adding to its anti-inflammatory role [18]. Additionally, lactoferrin acts as a competitor for bacterial LPS when binding to the CD14 receptor and, in this manner, it can attenuate the NF-κB-induced expression of various inflammatory mediators [19]. Upon tissue response to injury or infection, LF can control the oxidative burst of macrophages and neutrophils by iron sequestration. Thus, LF may play a role in the regulation of acute inflammation.

In vitro studies have shown that LF may act as a chemo-attractant not only for neutrophils, but also for antigen presenting cells (APCs), such as monocytes and dendritic cells (DCs), for which it also plays a role in activation and maturation [6]. Additionally, de la Rosa et al. demonstrated that the immunization of mice with ovalbumin in the presence of lactoferrin promoted Th1-polarized antigen-specific immune responses [6]. Lactoferrin has been reported as being able to modulate both cell-mediated and humoral immunity by helping in the maturation, differentiation and activation of T and B lymphocytes [2]. Therefore, we can conclude that LF participates both in the innate and adaptive immune responses, also by providing a link between the recognition of a pathogen and mounting the specific response.

## 3. Antiviral Activities of Lactoferrin

Lactoferrin can also bind to heparan sulfate proteoglycans (HSPGs), which are present on the cell surface and within the extracellular matrix, consisting of a core protein decorated with covalently linked glycosaminoglycan (GAG) chains [20]. HSPGs are expressed on the surface of almost all eukaryotic cell types. The binding between viruses and HSPGs usually occurs between closely packed basic amino acids present in viral surface proteins and called viral attachment ligands (VALs), and the negatively charged sulfated groups of heparan sulfate (HS), found in the glycocalix of the cell surface [21]. Heparan sulphate helps some viruses (for example, herpesvirus type 1, HSV-1, but also HIV) to establish contact between the virus particle and the cell surface, followed by rolling toward the virus’ specific receptor, fusing with the host membrane and the viral entry [22]. To date, the antiviral properties of LF have been confirmed by several in vitro studies [23]. Lactoferrin prevents the infection of the target cell of a broad spectrum of viruses, including both RNA and DNA viruses, enveloped as well as naked viruses (reviewed in [23]). 

**Figure 1:**
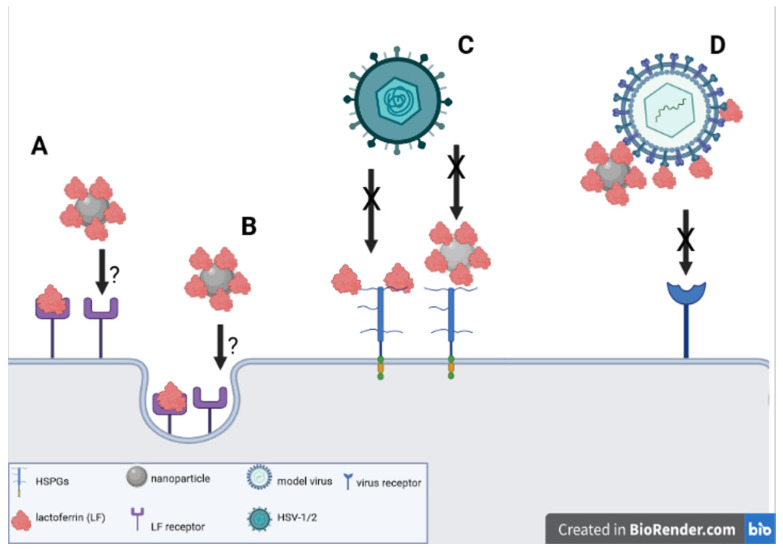
Schematic figure describing the possible mechanisms of antiviral actions by lactoferrin and lactoferrin-modified nanoparticles. (**A**) Lactoferrin can be internalized via endocytosis; the binding of lactoferrin-modified nanoparticles requires confirmation. (**B**) Endocytosis of lactoferrin-modified nanoparticles requires confirmation. (**C**) Lactoferrin or lactoferrin-modified nanoparticles can block HSV-1/2 infection through binding to heparan sulphate moieties present in the glycoproteins of the cell surface/extracellular matrix. (**D**) Lactoferrin or lactoferrin-modified nanoparticles can block the binding of a virus by binding to the virus attachment ligands (VALs), thus blocking the attachment of the virus to its specific receptor(s).

Lactoferrin can either interfere with heparan sulfate proteoglycans on the cell surface, bind directly to viruses or its receptors, or upregulate the antiviral response by the immune system (Figure 1). However, some authors also indicate its possible action through interfering with the endocytic pathway of virus infection. No significant differences in the anti-viral activity were found for both apolactoferrin and iron-bound hololactoferrin [23]. Interestingly, bovine lactoferrin (bLF) exhibits a higher antiviral activity than human lactoferrin (hLF), as shown for human papilloma virus (HPV), adenoviruses and human coronaviruses [23,24,25,26,27]. 

LF exerts antiviral activity against both HSV-1 and HSV-2, although it seems that the exact antiviral mechanism may be different, due to the difference of the viral proteins employed by each virus to entry into the target cells [25,26]. HSV initially binds to heparan sulfate of the host cells through the viral glycoprotein(s) gC or gB. In cells expressing either heparan sulfate or chondroitin sulfate or both, bLF strongly inhibited HSV-1 infection, but it was ineffective or less efficient in cells deprived of glycosaminoglycan or treated with glycosaminoglycan-degrading enzymes. This indicates that the anti-HSV-1 activity of lactoferrin depends on its interaction with the cell surface glycosaminoglycan chains of heparan sulfate and chondroitin sulfate (Figure 1) [27]. Human lactoferrin was demonstrated not only to inhibit the adsorption and entry of HSV-1 virions, but also cell-to-cell spread mediated by gD [28]. Concerning the in vivo antiviral activity of LF, the topical administration of 1% bLF decreased the infection of mouse cornea with HSV-1, but it did not influence general infection [29]. For HSV-2, the mechanisms of inhibition by hLF and bLF are slightly different [30]. The antiviral activity of bLF is independent of interaction between glycoprotein C (gC) and HS, as there was no difference between wild-type HSV-2 and its gC-truncated form. Furthermore, bLF also inhibited plaque-forming activity in cells devoid of GAG, which suggests that bLF interacts with HSV-2 receptor of non-GAG nature [30]. 

Waarts et al. used alphaviruses, adapted to interaction with HS, to test if LF can block infection by Sindbis virus (SIN) or Semliki Forest virus (SFV) [31]. Their results indicate that LF does not bind to the virus, but rather blocks HS on the cell surface, since bLF was able to block the infection of BHK-21 cells only with HS-adapted strains [31]. However, lactoferrin can inhibit viral attachment and penetration not only by binding to HS, but as for HSV-2, it can also bind to another receptor implicated in the virus infection. For example, Chien et al. demonstrated that bLF blocks Japanese encephalitis virus (JEV) infection by binding to HS and low-density lipoprotein receptor (LDLR), but not by binding to virus particles [32]. These findings further helped to find another receptor for non-HS-adapted strains of JEV [32]. The same group showed similar results for dengue virus (DENV), whose E protein interacts with highly sulfated glycosaminoglycans on the cell surface [33]. The authors demonstrated in vitro that bLF blocks DENV-2 entry into the cells by binding to HS, LDRL and dendritic cell-specific intercellular adhesion molecule 3-grabbing non-integrin (DC-SIGN), the latter being a surface receptor of dendritic cells, the antigen presenting cells (APC) [33]. Furthermore, the intracranial infection of suckling mice with DENV-2 premixed with bLF demonstrated a 60% reduction in mortality in comparison to mice infected only with DENV-2 [33]. 

Respiratory syncytial virus (RSV) is another virus employing heparan sulfate to initiate infection [34]. Lactoferrin directly interacted with the glycoprotein F(1) subunit of RSV and blocked further steps of in vitro infection [34]. Similarly, two envelope proteins of hepatitis C virus (HCV), E1 and E2, can bind both hLF and bLF in vitro [35]. The direct interaction between E2 and lactoferrin was demonstrated in vivo, by the coimmunoprecipitation of anti-human lactoferrin antibody with E2 protein [35]. Both bLf and hLf were demonstrated to inhibit adenovirus infection in a dose-dependent manner in vitro. Bovine LF showed stronger antiviral activity only when used for preincubation with epithelial cells or added during the attachment step [36]. The antiviral activity of bLf against adenoviruses is believed to be linked with the ability of bLF to interact with the adenovirus penton base (polypeptide III), the protein responsible for the virus binding to the integrin cell receptors [36].

Considering the fact that lactoferrin is naturally present in cervicovaginal lavage and increases upon bacterial vaginosis [37], it is not surprising that LF can block sexually transmitted viruses, such as HSV-2 [30], but also human papilloma virus (HPV) and HIV [24,38]. Heparan sulfate on the cell surface can also act as a receptor for HPV. HPV-16 virus-like particles (VLPs) incubated with HaCaT cells at the presence of bLF or hLF showed a dose-dependent inhibition of HPV-16 VLP binding to the keratinocyte cell surface [24]. The role of lactoferrin in protection from HIV infection was demonstrated by the role of lactoferrin gene polymorphism in the susceptibility to HIV-1 transmission from mother to newborns [38]. In vitro studies demonstrated that both bLF and hLF are potent inhibitors of HIV infection in vitro [39]. LF was shown to bind to the GPGRAF domain of the HIV gp120 glycoprotein [40]. It is well known that the binding of gp120 to the CD4 or chemokine receptors on the target cells is the basic mechanism responsible for the adsorption and entry of HIV into T cells. Therefore, it is possible that the main antiviral effect of LF against HIV is mediated in this manner (Figure 1) [40]. 

Coronaviruses are RNA viruses with a homotrimeric S (spike) glycoprotein, possessing N-linked glycans located on the envelope and comprising of two functional subunits (S1 and S2) in each spike monomer [41]. Homotrimers of S glycoproteins are involved in both host receptors binding (S1) and membrane fusion (S2) [41,42]. The main cell receptor for SARS-CoV is angiotensin-converting enzyme 2 (ACE2), but HSPGs have been recognized as supplementary binding sites for SARS-CoV-2 [41,42,43] and could play a role in the early attachment phase. Lang et al. demonstrated that LF could block the infection by SARS pseudovirus through binding to HSPGs, the latter providing the preliminary docking sites for the virus on the cell surface [43]. The study by Campione et al. demonstrated by in vitro and in silico studies that lactoferrin can attach directly to SARS-CoV-2 (spike S glycoprotein) and cell surface components, such as HSPGs [44]. These results were further partially confirmed by Hu et al., who demonstrated that both bLF and hLF show promising antiviral activity against multiple common human coronaviruses, including HCoV-OC43, HCoV-NL63, HCoV-229E as well as SARS-CoV-2 [45]. Taken together, these results suggest that LF can play a protective role in host defense against SARS-CoV-2 infection through binding to HSPGs on the cell surface, thus blocking the initial tethering of SARS-CoV-2 spike protein to host cell membrane (Figure 1). 

The influenza virus employs two surface glycoproteins, hemagglutinin (HA) and neuraminidase (NA), for the successful infection of the target cells. HA binds to the cell surface sialic acids and helps the virus to enter the target cells, while NA takes part in releasing the progeny virus from infected cells, acting as a hydrolase cleaving the glycosidic linkages of the sialic acid in glycoproteins and HA [46,47]. Further steps include receptor-mediated endocytosis and transfer to acidic late endosomes, where low pH triggers the HA-catalyzed release of viral ribonucleoprotein into the cytoplasm [46,47]. The work by Superti et al. (2019) [46] demonstrated that bLF blocked influenza virus infection through a specific interaction with the HA fusion peptide at a low pH. However, this effect was observed only during the early stage of infection and disappeared if bLF was added one hour after viral binding [46]. Lactoferrin, as the influenza virus, enters the cells by endocytosis and is found in endosome vesicles. Therefore, this work shows a completely different mechanism of LF antiviral action—not by interfering with the cell surface, but rather acting inside the cells at the early stage of infection. 

Furthermore, other authors suggested that the mechanism of anti-influenza activity of bLF relies on the sialylated glycans serving as receptor analogs to block IAV attachment to host cells at the early stage of viral infection [47]. On the other hand, Pietrantoni et al. (2012) [48] demonstrated that bLF inhibited influenza virus infection, if added directly after the adsorption step, independently from ion saturation or carbohydrate content [48]. Earlier work by Pietrantoni et al. (2010) [49] showed that bLF treatment inhibited apoptosis induced by the influenza virus by interfering with the function of caspase 3, an apoptosis effector, and bLF blocked nuclear export of viral ribonucleoproteins, thus preventing viral assembly [49]. This indicates that LF can interact with the intracellular pathways of virus entry; however, more research work is needed to elucidate the exact mechanism. 

Concerning pleiotropic activities of lactoferrin, it is worth remembering that this protein can chelate ferric ions, so it can thus influence both bacterial and viral replication and inhibit the formation of reactive oxygen species [26]. The disorders of iron homeostasis, induced by inflammation, increase intracellular iron concentration and favor viral replication [50]. The replication of viruses or infection-related activity of the innate cells (neutrophils and inflammatory macrophages) can lead to necrotic cell death, accompanied by the release of intracellular substances leading to the further development of inflammation [50,51]. Excessive inflammation at the site of infection can hinder the development of the immune response to pathogens, as observed in SARS-CoV-2 infection [52] but also in herpes simplex encephalitis [53]. Cutone et al. [54] demonstrated that bLF can cause a shift of polarized, pro-inflammatory M1 macrophages to anti-inflammatory M2 phenotype [54]. Activated M1 macrophages increase IL-6 production and downregulate proteins involved in iron metabolism [54]. bLF can downregulate IL-6 synthesis and upregulate ferropontin expression to regulate cell-to-blood iron export [54]. The ability of lactoferrin to reduce inflammatory reaction was also demonstrated in vivo for bacterial infections [55]. 

## 4. Nanoparticles Modified with Lactoferrin as Potent Antivirals

Nanomaterials are a diverse class of small-scale (<100 nm) objects (spheres, sheets and tubes), formed by physical or chemical methods and designed to offer unique physical and chemical properties. Recently, more and more data have been collected to describe possible biomedical applications of nanoparticles as microbicides. The use of nanoparticles as antiviral agents offers several advantages. Nanoparticles have the characteristics of a high surface-to-volume ratios, which enables to pack multiple antiviral agents onto the surface of nanoparticles. Therefore, it is possible to target specific biological sites in an active or passive manner by means of controlling hydrophobicity/lipophilicity, or the modification of nanocarrier surfaces with desired compounds. Furthermore, nanoparticles as nanocarriers are easily recognized and engulfed by immune cells, thus providing an opportunity to design vaccines that can mediate the targeted delivery of various antigens and adjuvants or immune regulatory agents in problematic cases encountered in classical vaccination approaches. 

Metallic nanoparticles (MeNPs) are relatively non-biodegradable, have rigid structures, and possess simple synthesis methodology. Based on its antimicrobial activity, silver has become one of the most prominent nanomaterials. Silver particles (AgNPs), having a long history of general use as an antiseptic and disinfectant, have been shown to be able to interact with the disulfide bonds of the glycoprotein/protein of microorganisms, such as viruses, bacteria and fungi [56]. However, the antiviral activity of unmodified AgNPs is none or very low—silver nanoparticles have to be modified in a manner that allows for interaction with the virus surfaces so that external double electric layer provides negative net charge. Baram-Pinto et al. (2009) [57] developed silver nanoparticles that were modified with mercaptoethane sulfonate (Ag-MES) [57]. The MES-modified AgNPs of 4 nm mimicked well the heparan sulfate present on the host cell and blocked HSV-1 attachment to the cells [57]. In the same manner, sulfonate-modified AuNPs inhibited the entry of the virus into the cells and prevented the cell-to-cell spread of HSV-1 [58]. While gold nanoparticles are inert and non-toxic, AgNPs are known to release silver ions, which can further influence cellular metabolism [59,60]. Although the release of silver ions adds to antiviral activities, it has to be taken into account during the development of modified AgNPs. Furthermore, the toxicity of AgNPs and, to a lesser extent, that of AuNPs, depend on their size with small nanoparticles < 10 nm, being able to directly cross the cellular membrane. On the other hand, nanoparticles sized >20 nm are actively internalized by cells in the process of endocytosis [61]. Park et al. postulated the so-called the Trojan Horse mechanism of AgNP cytotoxicity, involving the release of silver ions from the surface of NPs in acidified compartments. The released ions induced reactive oxygen species (ROS) production in mitochondria, further triggering the activation of immune cells through stress signals and secreted cytokines (i.e., TNF-α) [62]. 

Our group described the anti-HSV activity of tannic-acid-modified AgNPs (TA-AgNPs) by using both in vitro and in vivo models of HSV-1 and HSV-2 infections [63,64]. The mechanisms of the antiviral action of TA-AgNPs included the blocking of virus attachment, entry as well as the induction of anti-viral cytokine and chemokine production. Additionally, TA-AgNPs turned out to be good activators of immune response, since they were also able to overcome the inhibition of DC maturation by live or inactivated HSV-2, normally observed in this model of infection [61,64]. Therefore, we showed that tannic-acid-modified AgNPs can act both as an effective microbicide and a novel class of nano-adjuvants. 

Nanoparticles can interact with proteins, forming the so-called interfacial protein corona, which imparts specific biological activity to nanoparticles. The work by Nayak et al. demonstrated that bLF can be absorbed onto the AgNP surface by van der Waals interactions and hydrogen bonds, without influencing the protein conformation and stability [65]. BLf also helped to decrease the AgNPs-mediated cytotoxicity [65]. We also demonstrated that pretreatment with human lactoferrin functionalized gold and silver nanoparticles sized 10 or 30 nm prevented HSV-2 infection by the direct inhibition of virus attachment and the penetration and blocking of infection (Figure 1) [66]. Lactoferrin used in the same concentrations did not show significant antiviral effects, particularly when used in vitro. We propose that lactoferrin in nanometal conjugates binds to HSPGs on the cell surface in a much more effective manner, creating in this manner a barrier preventing HSV-2 infection. The results of in vivo experiments showed that, while all tested lactoferrin conjugates were very effective in decreasing HSV-2 titers during vaginal infection in comparison to lactoferrin itself (though only early during infection), the LF conjugates showed an additional immunomodulatory effect [66]. All LF nanoparticles induced a significant increase in antiviral cytokines and chemokines in the vaginal tissue [66], followed by the influx of antigens presenting cells (dendritic cells). As previously shown for tannic-acid-modified NPs, LF conjugates provided additional adjuvant-like activity most probably induced by nanoparticles themselves [61,64,66]. Since the adjuvant activity of nanoparticles depends strongly on their physical and chemical properties, certain caution should be taken when planning in vivo treatment with AgNPs sized <10 nm. In our experiments, the vaginal tissued treated with 10 nm LF-AgNPs showed a significant increase in TNF-α and IL-1β [66]. While these two cytokines are important for mounting antiviral response to HSV-2, they are also pro-inflammatory cytokines. The excessive production of proinflammatory cytokines can lead to the amplification of the inflammation induced by the virally infected cells and further result in a delayed or ineffective antiviral response [67]. 

**Figure 2:**
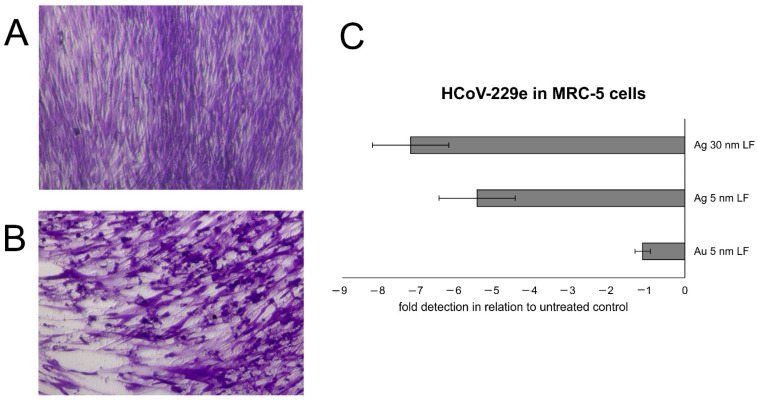
Human epithelial MRC-5 cells (**A**) uninfected and (**B**) infected with HCoV-229e at 48 h post infection. (**C**) HCoV-229e titers in MRC-5 cells at 48 h, treated with 7.5 μg/mL of lactoferrin modified 5 nm AuNPs, 5 nm AgNPs or 30 nm AgNPs. Data represent means ± SEM (Own unpublished data).

Additionally, we tested hLF-modified gold and silver nanoparticles sized 10 or 30 nm against human coronavirus—HCoV-229e in vitro in MRC-5 cells (Figure 2). We mixed modified nanoparticles at non-toxic concentrations, determined earlier, incubated for 1 h, then applied to MRC-5 cells and incubated for 48 h. After this time, cells were harvested and the titers of HCov-229e were accessed using commercial qPCR probe Vi06439671_s1 (Thermo Fisher Scientific, Waltham, MA, USA). Our results demonstrated that the inhibition of HCoV-229e infection was metal and size-dependent with LF-modified 30 nm AgNPs showing the most promising anti-viral activity (Figure 2). 

In conclusion, our experiments prove that lactoferrin-modified nanoparticles of noble metals (silver and gold) can be considerate as very promising new class of antivirals (Figure 1).

## 5. Lactoferrin and Nanotechnology

Lactoferrin, as other biologically active compounds, is being combined with nanoparticles as new carriers or new formulation. The combination of lactoferrin with nanoparticles is usually used to prepare a new combination of highly toxic drugs. On the other hand, we can observe this type of lactoferrin connections for use in functional foods, supplements and of course in medicine. We can recognize some kinds of combination and formulations.

### 5.1. Lipid-Based Nanocarriers (Emulsion)

The sol–oil or emulsion (water-in-oil) method is one of those methods in which lactoferrin in aqueous solution is combined with olive oil. The process takes place under conditions of 4 °C with sonication and then freezing in liquid nitrogen. This process was used to prepare anti-cancer substances in order to obtain a formulation with a lower toxicity [68,69]. Another type of emulsion was encapsulation lactoferrin with antiviral particles (Zidovudine) [70]. Similar results were obtained with comparable encapsulation conditions. The nanoparticles were prepared at the size of 50–60 nm with a yield of 67% and a good physical stability at room temperature and 4 °C. Oral-use Zidovudine and its lactoferrin complex showed similar activity against HIV-1 [70]. In addition, the new drug formulation displayed an improved pharmacokinetic profile and a lower organ toxicity [70]. Methodologically, it is possible to prepare various forms and degrees of encapsulation, size and stability. Single-, double- and triple-loaded drugs with nanoparticles with lactoferrin can be produced. In this case, lactoferrin acts as an emulsifier.

Another type of encapsulation depends mainly on the presence of an oily phase in which the drug is soluble (with the organic solvent). In the next step, the oily phase is slowly added to the lactoferrin-containing water phase. The core of the emulsion is formed by subjecting this mixture to shear forces at a low homogenization rate. The mixture can then be sonicated for approximately 2 min. The end product can be obtained by removing the residual organic solvent on a rotary evaporator [71]. In the same way, an insoluble drug can be prepared with nanoparticles bound to albumin and lactoferrin, for example, paclitaxel [72]. This is a very good case for encapsulating delivery systems highly lipophilic drugs in protein nanoparticles [73].

### 5.2. Inorganic Nanoparticles

A very important use of lactoferrin is its conjugation to inorganic nanoparticles. The conjugates have special features: higher loading, easiness of connection with ligands and biocompatibility. LF-coated magnetic nanocarriers can be used for cancer diagnosis and therapy, acting as contrast agents in magnetic resonance imaging (MRI) [74,75,76]. The most interesting are superparamagnetic iron oxide nanocarriers (Fe_3_O_4_-LF). Iron oxides are prepared by the coprecipitation of Fe^2+^ and Fe^3+^ in the presence of a base. The size, shape and composition of iron NPs depend on the type of the salt used, Fe^2+^ to Fe^3+^ ratio, pH and ionic strength [77,78]. Magnetic nanocarriers can be combined with lactoferrin and some co-ligands, e.g., via 1-Ethyl-3- (3-dimethylaminopropyl) carbodiimide (EDC), N-hydroxysuccinimide (NHS), polyvinyl alcohol (PVA) and polyacrylic acid (PAA) [79]. Upon mixing, the LF and co-ligand nanoparticles can form an amide bond between the primary amine and the carboxyl group of the second coupling reagent. The formation of a stable amide bond relies on the activation of the LF carboxyl groups by the coupling reagent [80,81]. Another group of nanocarriers includes silica NPs composed of silicon dioxide of a vast range of sizes. SiNPs were approved by the FDA because they are considered safe [82]. Silica nanoparticles can be a very useful smart brain delivery system and they have been used for the efficient delivery of drugs without damaging the BBB (blood–brain barrier) function. The lactoferrin-based system was based on covalently binding LF to silica nanoparticles (SiNPs). In the next step, LF-SiNPs were PEGylated to prolong their blood circulation [83].

### 5.3. Polyamidoamine PAMAM Dendrimers

Dendrimers are highly branched radially symmetrical three-dimensional structures with many functional groups that make it easy to conjugate with ligands, such as LF. The most popular dendrimers are fabricated from polyamidoamine (PAMAM) [84]. Due to the possibility of aggregation of platelets, the most beneficial in medicine are first, second and third generations. The most common combination of lactoferrin with dendrimers is PAMAM dendrimer, polyethylene glycol (PEG) and lactoferrin (PAMAM—PEG—LF). In this formula, PEG was used as a spacer for targeted brain delivery [85,86]. In another study, LF was covalently coupled to PAMAM-PEG. The purpose of designing such a use for lactoferrin was a non-viral gene therapy in Parkinson’s disease. On the other hand, the preparation of this type of dendrimer combinations is focused on the preparation of nanoparticles in the form of an emulsion and consists of mixing the appropriate amounts of substrates [87,88].

## 6. Conclusions

The use of lactoferrin conjugates with nanoparticles seems to offer new solutions for the improvement of the anti-viral activity of lactoferrin itself (Figure 1). We can speculate that the 3D structure of lactoferrin conjugates with nanoparticles facilitates the interaction with both cellular targets—HSPGs as well as with VALs. The exact mechanism of the interaction of virus/cells and lactoferrin functionalized nanoparticles requires further research with the use of techniques allowing the visualization of this interaction, such as TEM and cryoTEM. Furthermore, tests employing different viruses interacting with HSPGs are also required to be performed both in vitro and in vivo in animal models. Last, but not least, pharmacological formulations need to be developed and tested for their efficacy, safety and stability. 

## Data Availability

Data available upon request.
